# Hyperbaric Oxygen Therapy is Ineffective as an Adjuvant to Daptomycin with Rifampicin Treatment in a Murine Model of *Staphylococcus aureus* in Implant-Associated Osteomyelitis

**DOI:** 10.3390/microorganisms5020021

**Published:** 2017-04-25

**Authors:** Nis Pedersen Jørgensen, Kasper Hansen, Caroline Marie Andreasen, Michael Pedersen, Kurt Fuursted, Rikke L. Meyer, Eskild Petersen

**Affiliations:** 1 Department of Infectious Diseases, Aarhus University Hospital, 8200 Aarhus, Denmark; eskild.petersen@clin.au.dk; 2 Department of Clinical Microbiology, Aarhus University Hospital, 8200 Aarhus, Denmark; 3 Comparative Medicine Lab, Department of Clinical Medicine, Aarhus University Hospital, 8200 Aarhus, Denmark; kasperhansen@bios.au.dk (K.H.); michael@clin.au.dk (M.P.); 4 Department of Rheumatology, Aarhus University Hospital, 8200 Aarhus, Denmark; c.m.andreasen@clin.au.dk; 5 Microbiology and Infection Control, Statens Serum Institut, 2300 Copenhagen, Denmark; kfu@ssi.dk; 6 Interdisciplinary Nanoscience Center (iNANO), Aarhus University, 8000 Aarhus, Denmark; rikke.meyer@inano.au.dk; 7 Department of Bioscience, Aarhus University, 8000 Aarhus, Denmark

**Keywords:** Implant-associated osteomyelitis, hyperbaric oxygen therapy, biofilm, *Staphylococcus aureus*, bone turnover, inflammation

## Abstract

Implant-associated infections caused by bacterial biofilms are difficult to treat. Surgical intervention is often necessary to cure the patient, as the antibiotic recalcitrance of biofilms renders them untreatable with conventional antibiotics. Intermittent hyperbaric oxygen treatment (HBOT) has been proposed as an adjuvant to conventional antibiotic treatment and it has been speculated that combining HBOT with antibiotics could improve treatment outcomes for biofilm infections. In this study we addressed whether HBOT could improve treatment outcomes of daptomycin and rifampicin combination therapy. The effect of HBOT on the treatment outcomes of daptomycin and rifampicin against implant-associated osteomyelitis was quantified in a murine model. In total, 80 mice were randomized into two groups receiving antibiotics, either alone or in combination with daily intermittent HBOT (304 kPa for 60 min) following injection of antibiotics. Treatment was initiated 11 days after animals were infected with *Staphylococcus aureus* and treatment duration was 14 days. We found that HBOT did not improve the cure rate and did not reduce the bacterial load on the implant surface or in the surrounding tissue. Cure rates of daptomycin + rifampicin were 40% in infected tibias and 75% for implants while cure rates for HBOT-daptomycin + rifampicin were 50% and 85%, respectively, which were not significantly higher (Fisher’s exact test). While it is encouraging that the combination of daptomycin and rifampicin is very effective, our study demonstrates that this efficacy cannot be improved by adjuvant HBOT.

## 1. Introduction

The incidence of implant-associated osteomyelitis (OM) is increasing. There are approximately 100,000 cases of implant-associated OM each year in USA, with an infection rate of 5% to 15% in fracture-fixation devices and 0.3% to 1% in joint-prosthesis [1,2,3,4,5]. The incidence rate of implant-associated OM is site dependent, and for prosthetic hip infection, there was no decrease in the 1-year infection rate from 2005 to 2014 [6]. *Staphylococcus* are the most common bacteria isolated in implant-associated OM, with *Staphylococcus aureus* accounting for 35% [1]. Biofilms are communities of sessile bacteria enclosed in a self-produced extracellular matrix. Biofilms exhibit great resistance to the host immune response and are highly recalcitrant to antimicrobial treatment [2,3]. This recalcitrance is partly caused by impaired penetration of antibiotics through the extracellular matrix, and by oxygen gradients that lead to oxygen depletion a low metabolic rate of bacteria in the biofilm [7,8,9,10]. Current treatments rely on combination antibiotic therapy or swift surgical intervention with removal of infected foreign material [11,12]. Expenditures associated with treating implant-associated OM are expected to dramatically increase in the coming years, and since certain patients cannot undergo surgical therapy, a non-invasive treatment alternative is urgently needed [4,13,14].

As clinicians search for effective ways for combating biofilm infections, The European Society of Clinical Microbiology and the Infectious Diseases Study Group on Biofilms have called for research projects on the possible benefits of hyperbaric oxygen treatment (HBOT) in treatments of biofilm infections [15]. HBOT has historically been proposed as a treatment option for chronic osteomyelitis [16,17,18]. The majority of data to support this proposal comes from experimental studies performed in the late 1970s and early 1980s, before the impact of biofilm formation on chronic infections was fully appreciated. Models of medullary OM demonstrated that the effect of HBOT alone is comparable to that of cephalotin [16]. Another study demonstrated that a combination of cefazolin and HBOT was superior to cefazolin alone [19]. However, a more recent study demonstrated that HBOT could stimulate bacterial growth and thereby accelerate the infection. This was shown by Shandley et al. who used a model of implant-associated OM to test the effect of HBOT (100% O_2_ at 240 kPa for 80 min) [20]. They found that the bacterial load of Methicillin-resistant *Staphylococcus aureus* (MRSA) in infected tibias was significantly higher in HBOT-treated animals compared to untreated animals, concluding that HBOT accelerates the growth of *S. aureus* [20].

HBOT is most commonly defined as an inhalation of 100% oxygen at pressures exceeding the normobaric pressure of 101.3 kPa found at sea level [21]. Breathing air at normobaric pressure leads to a partial oxygen pressure (PO_2_) of roughly 100 mmHg (13.33 kPa) in arterial blood (PaO_2_) and approximately 55 mmHg (7.33 kPa) in certain tissues [18]. However, increasing the ambient oxygen pressure to approximately 304 kPa, e.g., in a pressure chamber, results in a PaO_2_ of roughly 2000 mmHg (266.64 kPa), with tissue PO_2_ around 500 mmHg (66.66 kPa) [18]. In a rabbit model of OM, the O_2_ tension of infected bone was measured as 234.1 ± 116.3 mmHg (31.21 ± 15.5 kPa) at a oxygen pressure of 300 kPa, i.e., approximately 14 times higher than the corresponding normobaric situation [17]. HBOT triggers several mechanisms that make HBOT an interesting candidate in treatments of various infections. The production of reactive oxygen species is elevated, leading to increased oxidization of proteins, leading to inhibition of bacterial metabolism and stimulated oxygen-dependent intracellular killing in phagocytes [22,23,24]. An in vitro study demonstrated that increasing the oxygen pressure stimulated neutrophil-mediated killing of *S. aureus* in a dose-dependent fashion, with a maximum effect at the highest oxygen tension tested (20.0 kPa) [25].

In the context of treating biofilm infections, HBOT is a potentially effective adjuvant to antibiotic therapy because introduction of oxygen to the otherwise oxygen-depleted parts of the biofilm could stimulate metabolic activity and thereby restore antibiotic susceptibility of the bacteria. This has been shown for *Pseudomonas aeruginosa* biofilms in vitro [26].

As the majority of studies done on *S. aureus* OM has been performed in a model without an implant or with antibiotic regimens that are no longer considered gold standard therapy for implant-associated OM, we were interested in whether adjuvant HBOT could increase the efficacy of current gold standard antibiotic therapy and a combination regimen containing rifampicin [12]. Whether HBOT has a role in the treatment of biofilm infections is currently unclear, as the older studies demonstrated a beneficial effect of HBOT, while one recent study, using a model of implant-associated OM, yielded results that contradict the previous findings, as HBOT alone stimulated bacterial growth [20].

The aim of this study was to determine if HBOT could improve the treatment outcome of implant-associated OM when used as an adjuvant to antibiotic combination treatment with daptomycin and rifampicin because this treatment combination is very effective against *S. aureus* biofilm infections in animal studies [27,28,29,30,31,32].

## 2. Materials and Methods

### 2.1. Isolate

A batch of *Staphylococcus aureus* ATCC12600 was thawed from −80 °C, plated on 5% blood agar plates (SSI Diagnostic, Hillerød, Denmark) and incubated overnight at 37 °C. From this batch, colonies were re-plated on 5% blood agar plates and incubated overnight at 37 °C. Finally, bacteria were suspended in 3% Tryptic Soy Broth (TSB) at a concentration of 1.5 × 10^6^ colony forming units (CFU) per mL. Minimum inhibitory concentration (MIC) for daptomycin were 0.38 mg/L and 0.008 mg/L for rifampicin and the minimum biofilm eradication concentration (MBEC) value for daptomycin was 1024 mg/L as previously reported [33].

### 2.2. Generation of Pathogenic Challenge

Steel implants (Benfidan, Nykøbing Mors, Denmark) with a diameter of 0.25 mm were sterilized and placed in 5 mL of 25% murine serum +75% phosphate buffered saline (PBS) at 37 °C for 24 h. Implants were then submerged in the prepared *S. aureus*-enriched TSB media for 24 h at 37 °C.

### 2.3. Murine Model

#### 2.3.1. Study Design

A total of 80 female C57Bl6/j mice were divided into two cohorts of 40 mice. Each cohort was further divided into two groups of 20 mice. In both cohorts there was a treatment group receiving daptomycin + rifampicin and a control group received NaCl. Antibiotics were administered 120 min prior to HBOT. Cohort one received daily HBOT for 14 consecutive days (see HBOT protocol), while cohort two was handled similarly to cohort one, although retained from HBOT.

#### 2.3.2. Experimental Murine Model

The animal model of implant-associated osteomyelitis is described in detail elsewhere [34]. In brief, anesthetized C57Bl6/j female mice, aged 8–10 weeks received a transcortical tibia implant. The implant was incubated in TSB media containing *S. aureus* ATCC 12600 at an initial inoculum of 1 × 10^6^ CFU/mL for 24 h at 37 °C prior to insertion. After 11 days of observation, during which body mass, activity, and food intake were monitored, animals were subdivided into groups of 20 animals.

The study was approved by the Animal Research Inspectorate under the Danish Ministry of Justice, permission numbers 2012-15-2934-00716 and 2013-15-2934-00884.

### 2.4. Animal Welfare

The body mass of all animals was assessed daily and the overall welfare was judged according to protocolled humane endpoints (weight loss more than 15%, severe lethargy, loss of fur). Prior to euthanasia, cage identification was blinded and a qualitative assessment of abscess formation on the infected tibia was performed. Gross deformation was awarded “++”, inflammation signs (redness, swelling, tenseness) was awarded “+”, and normal phenotype was awarded “−”. The animal’s contralateral limb served as internal control. Following euthanasia 48 h following last treatment, tibias and implants were extracted and analysed.

### 2.5. Protocol for In Vivo Hyperbaric Oxygen Therapy

Animals of Cohort 1 received one daily HBOT during 14 consecutive days in a custom-built 60 L steel pressure chamber. Ten mice were placed together in well-ventilated cages, with three cages inside the pressure chamber during HBOT. CO_2_-removal was ensured using soda lime CO_2_-scrubbers inside the pressure chamber and by continually flushing the chamber with 10 L/min oxygen. Each day, the pressure chamber was pre-flushed at normobaric pressure with 15 L/min 100% oxygen for 10 min, before a 10 min linear 20 kPa/min pressurization (i.e., to 304 kPa) was initiated. After 60 min the pressure chamber was decompressed linearly at a rate of 20 kPa/min. Control groups were handled analogously to HBOT animals but were not pre-flushed with oxygen and not pressurized. During HBOT, animals could be observed through transparent glass in the pressure chamber, and no apparent adverse events of HBOT were observed (e.g., oxygen toxicity seizures or apparent stress full behaviour). Operation of the pressure chamber was done manually, and at all times, chamber pressure was visible via pressure gauge. Prior to each HBOT session, pressure equipment was calibrated.

### 2.6. Antibiotic Treatment

Daptomycin (Novartis, Basel, Switzerland) 50 mg/kg daily and rifampicin (Novartis, Basel, Switzerland) 25 mg/kg daily [27,35] were used. Control groups were administered 0.1 mL of 0.9% NaCl daily. All administrations were subcutaneous. Prior to initiation of treatment, the infection rate was 100% (all animals had subcutaneous abscess formation adjacent to the implant site). At the end of the study period, all animals were euthanized by cervical dislocation under general isoflurane (Baxter, Deerfield, IL, USA) anaesthesia. Prior to euthanasia, while generally anesthetized, blood samples were obtained by intracardial aspiration. Bone homogenization and implant sonication with subsequent serial dilution and culturing was performed immediately following either sonication or homogenization as previously described [34]. Treatment duration was 14 days and the animals were euthanized 48 h after the final antibiotic administration.

### 2.7. Post Mortem Analysis

Following euthanasia, implants and tibias were extracted as described previously [34]. In addition, the following analysis was included to increase the bacterial detection limit, based on a study performed by Metsemakers et al. [36]. Following sonication, each implant was placed in 1 mL of 3% TSB and incubated for 48 h at 37 °C. As the detection limit on the agar-based method is 100 CFU/implant, implant samples that were negative on agar plates, but positive in the TSB media were given the standardized value of 10 CFU/implant. Samples with no growth in either TSB or on agars were considered sterile.

Spleens were placed in 1 mL PBS and subjected to same homogenization protocol as the tibia samples, described in [34]. Afterwards serial dilutions were performed and plated on 5% blood agar. Plates were read following incubation for 24 h.

### 2.8. Serologicalbone Formation and Resorption Biomarkers

As the tibias from all animals were homogenized to measure primary outcome, we included serological biomarker measurement to address whether HBOT would affect either osteoclast or osteoblast activity. Plasma was separated from whole blood. Tartrate-resistant acid phosphatase 5b (TRAP 5b), a bone resorption marker specific to osteoclasts, was determined using a mouse TRAP 5b enzyme-linked immunosorbent assay [37]. Procollagen type 1 amino-terminal propeptide (P1NP), a marker of bone formation, was determined using an enzyme immunoassay [37]. Assays were performed in accordance with the manufacturer’s recommendations (Immunodiagnostic Systems Nordic, Copenhagen, Denmark) and analysed on a Thermo Scientific Multiscan Go (Thermo Fisher Scientific, Waltham, MA, USA) microplate spectrophotometer.

### 2.9. Statistical Analysis

Non-parametric tests were used, as the data were not normally distributed. Mann–Whitney’s test was used to compare differences. To test the dichotomous outcomes of abscess recession and ostensible “cure rate” Fisher’s exact test was used. All statistic analysis was performed with GraphPad Prism version 5.0f software (GraphPad Software, CA, USA).

## 3. Results

### 3.1. HBOT Leads to Initial Loss in Body Mass, but Animals Regain Mass within Days

**Figure 1:**
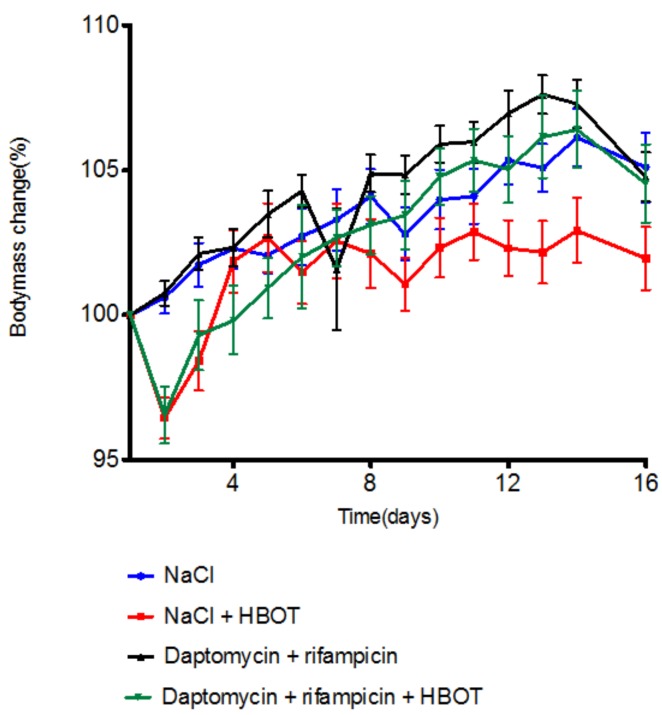
Mean mouse body mass ± standard deviation (SD) (% of body mass at day 1) throughout study period (*n* = 20 per group). Hyperbaric oxygen treatment (HBOT) animals experienced an immediate reduction in body mass of from 2% to 3% following initiation of HBOT. The body mass of mice treated with NaCl + HBOT (square) reached starting mass (100%) by day 4. The body mass of daptomycin + rifampicin + HBOT-treated mice (inverted triangle) stabilized at 100% by day 6. By the end of the study, all groups had a mean body mass above the mean body mass at the commencement of the study.

As HBOT can lead to adverse effects (primarily acute reactions with seizures or pulmonary oedema), animals were observed during HBOT, and body mass was measured daily. On day 2 of HBOT, the HBOT-cohort experienced a 3 to 4% body mass reduction, while the body mass of the ambient cohort remained stable (Figure 1). By day 4, the mass of the NaCl + HBOT group had stabilized at 100% of initial body mass, while the daptomycin + rifampicin + HBOT group reached 100% of initial mass by day 5. The body mass of the NaCl + HBOT group stagnated around 100% of initial body mass, while the remaining three groups steadily gained mass, and the mass at the end of the study was approximately 105% for all three groups. All mice, in both cohorts, survived until the end of the study, but the mice in the NaCl group were notably lethargic compared to the mice in the other groups.

### 3.2. HBOT Treatment Reduces Abscess Signs

All animals developed a subcutaneous abscess on the tibia containing the infected implant by day 11 following surgery. At the end of the treatment period, prior to euthanasia, animals were inspected and abscess recession was evaluated. NaCl + HBOT significantly reduced the number of animals with abscess signs (+/++) compared to NaCl (Table 1) (*p* = 0.047 Fisher’s exact test). The effect of NaCl + HBOT and daptomycin + rifampicin + HBOT was comparable in reducing abscess signs (*p* = 0.731, Fisher’s exact test).

### 3.3. HBOT Treatment Does Not Improve the Outcome from Antibiotic Therapy

**Figure 2:**
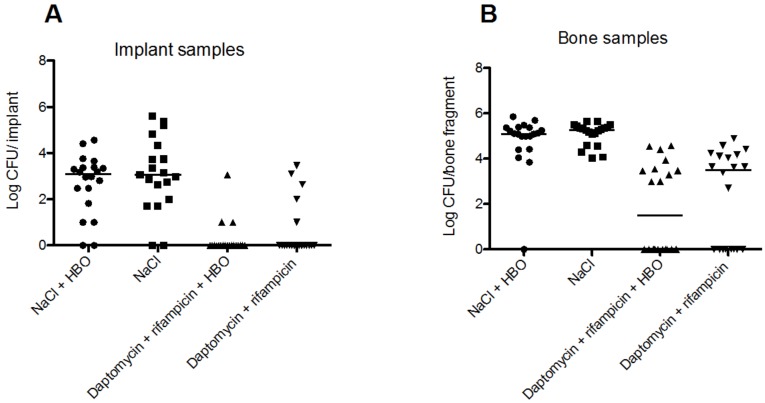
**A:** Median bacterial load of implants for NaCl group was 3.06 (0.0–5.59) log colony forming units (CFU)/implant (median, range), while the NaCl + HBOT group had a bacterial load of 3.08 (0.0–4.56) log CFU/implant, which was not significantly lower (group *n*-value = 20, *p* = 0.54, Mann–Whitney test). Median bacterial load for daptomycin + rifampicin was 0.0 (0.0–3.46) log CFU/implant, which was no different than for daptomycin + rifampicin + HBOT, 0.0 (0.0–3.06) log CFU/implant (group *n*-values = 20, *p* = 0.37, Mann Whitney test). In total, 17/20 (85%) and 15 (75%) of the implants in the daptomycin + rifampicin + HBOT and daptomycin + rifampicin groups, respectively, were without growth. There was no significantly difference in cure rates between the two treatment groups (group *n*-value = 20, *p* = 0.685, Fisher’s exact test). Both treatments resulted in a significant reduction of bacterial load compared to both NaCl or NaCl + HBOT (*p* < 0.0001, Mann Whitney test). **B:** Infected bones of the NaCl-treated animals had a bacterial load of 5.27 (4.03–5.88) log CFU/bone (median, range), while the NaCl + HBOT group had a median load of 5.1 (0.0–5.86) log CFU/bone, which was not statistically different from NaCl-treated animals (*n*-value = 20, *p* = 0.34, Mann–Whitney test). Daptomycin + rifampicin treatment eradicated infection in 8/20 samples (40%) and the median bacterial load in uncured bones was 3.51 (0.0–4.89) log CFU/bone, while daptomycin + rifampicin + HBOT eradicated infection in 10/20 samples (50%) and reduced the bacterial load in the uncured bones to 1.5, (0.0–4.59) log CFU/bone. There was no difference in “cure rate” (*p* = 0.751, Fisher’s exact test) or in mean bacterial load (*p* = 0.31, Mann Whitney test).

HBOT did not improve the outcome of antibiotic treatment, measured through the bacterial load on implants and in bones at the end of the treatment period (Figure 2). The median bacterial load in the NaCl group was 3.06 (range: 0.0–4.56) log CFU/implant and in the NaCl+ HBOT group 3.08 (range: 0.0–5.60) log CFU/implant. The difference was not significant (*p* = 0.54, Mann–Whitney test) (Figure 2A). Treatment with daptomycin + rifampicin reduced median bacterial load to 0.0 (range: 0.0–3.46) log CFU/implant, while the daptomycin + rifampicin + HBOT treatment reduced the bacterial load to 0.0 (range: 0.0–3.01) log CFU/implant. The difference in mean values of the two treatment groups was not significant (*p* = 0.37, Mann–Whitney test). In total, 17/20 (85%) of the implants in the daptomycin + rifampicin + HBOT group were sterile, while 15 (75%) of the implants in the daptomycin + rifampicin were sterile. This ostensible cure rate was not higher in the daptomycin + rifampicin + HBOT group (*p* = 0.685, Fisher’s exact test). Both treatments resulted in a significant reduction of bacterial load compared to the NaCl and NaCl–HBOT groups (*p* < 0.0001, Student’s *t*-test).

HBOT did not affect the bacterial load in infected bones (Figure 2B). Animals treated with NaCl + HBOT had a median load of 5.1 (range: 0.0–5.86) log CFU/bone, which was not significantly different from the NaCl-treated control animals (5.27, range: 4.03–5.88 log CFU/bone, *p* = 0.34, Mann–Whitney test). Daptomycin + rifampicin treatment eradicated the infection in 8/20 samples (40%), while daptomycin + rifampicin + HBOT eradicated the infection in 10/20 samples (50%). In uncured bones the median bacterial load was 3.51, range: 0.0–4.89 and 1.5, range: 0.0–4.59 log CFU/bone, respectively. There was no difference in “cure rate” (*p* = 0.751, Fisher’s exact test) or in median bacterial load (*p* = 0.31, Mann Whitney test).

### 3.4. HBOT Leads to Elevated Bone Turnover in Animals Treated with Antibiotics

**Figure 3:**
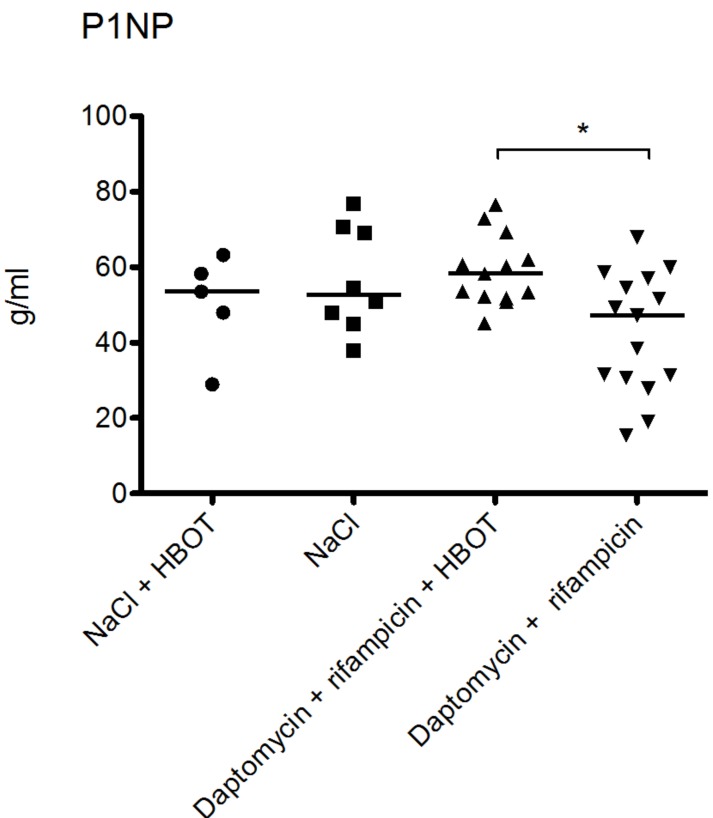
Procollagen type 1 amino-terminal propeptide (P1NP) levels were comparable in the two groups treated with NaCl. In the NaCl + HBOT group the median concentration was 53.53 g/mL (range: 28.98–63.24 g/mL), while the median concentration was 52.65 g/mL (range: 37.94–76.92 g/mL) in the NaCl group. There was no significant difference between these two groups (*p* = 0.62 Mann Whitney’s test). HBOT resulted in slightly elevated P1NP levels in animals treated with daptomycin + rifampicin. Median P1NP concentration was 58.25 g/mL (range: 45.25–76.55 g/mL) for the daptomycin + rifampicin + HBOT group compared to 47.11 g/mL (range: 15.30–67.86 g/mL) in the daptomycin + rifampicin group. * marks significant statistical difference (*p* = 0.0086, Mann Whitney’s test).

**Figure 4:**
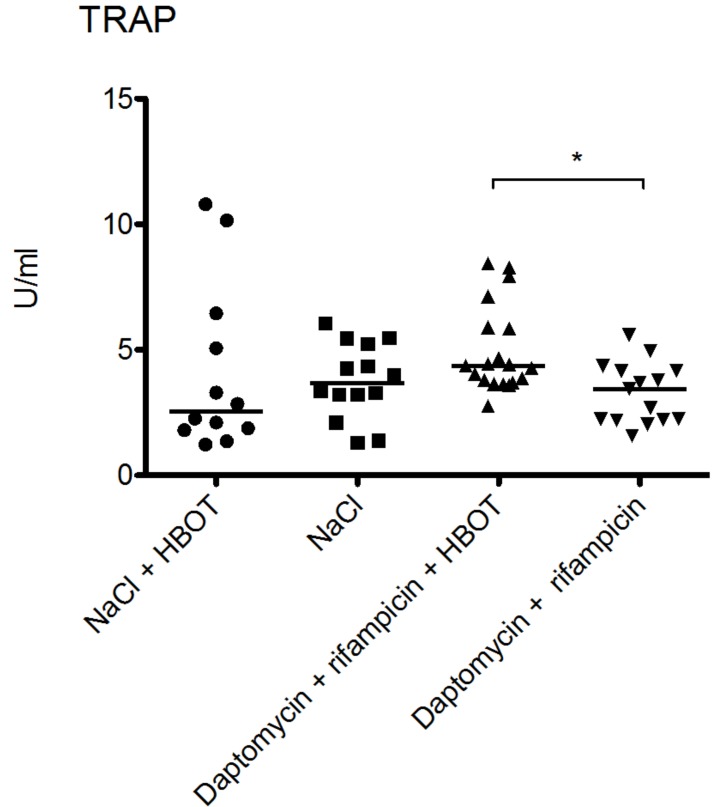
The tartrate-resistant acid phosphatase 5b (TRAP 5b) levels were comparable in both groups treated with NaCl. The median value for NaCl + HBOT was 2.549 U/mL (range: 1.230–10.79 U/mL), while median value for the NaCl group was 3.289 U/mL (range: 1.296–6.041 g/mL). Hyperbaric oxygen therapy resulted in a slight elevation of TRAP 5b levels in animals treated with daptomycin + rifampicin. The median TRAP 5b value was 2.770 U/mL (range 4.363–8.441 U/mL) in the daptomycin + rifampicin + HBOT group, which was higher than the median value in the daptomycin + rifampicin group of 1.577 U/mL (range 3.446–5.589 U/mL). * marks significan statistical difference (*p* = 0.005, Mann–Whitney’s test).

Intermittent HBOT resulted in elevation of bone turnover in animals treated with daptomycin and rifampicin (Figure 3 and Figure 4). Median P1NP concentration was 58.3 g/mL (range: 45.2–76.5 g/mL) for the daptomycin + rifampicin + HBOT group compared to 47.1 g/mL (range: 15.3–67.9 g/mL) in the daptomycin + rifampicin group. This difference was statistically significant (*p* = 0.0086, Mann–Whitney test). However, HBOT did not significantly affect the P1NP levels in animals receiving NaCl. In the NaCl + HBOT group, median concentration was 53.5 g/mL (range: 28.9–63.2 g/mL), while median concentration was 52.6 g/mL (range: 37.9–76.9 g/mL) in the NaCl group.

TRAP 5b levels were higher in daptomycin + rifampicin + HBOT treated animals compared to the daptomycin + rifampicin group. The median TRAP 5b value was 2.77 U/mL (range 4.36–8.44 U/mL) in the daptomycin + rifampicin + HBOT group, which was significantly higher than the median value in the daptomycin + rifampicin group, which was 1.58 U/mL (range 3.45–5.59 U/mL) (*p* = 0.005, Mann–Whitney’s test). TRAP 5b levels were comparable in the two NaCl groups. The median value for NaCl + HBOT was 2.55 U/mL (range: 1.23–10.79 U/mL) while the median value for the NaCl group was 3.299 U/mL (range: 1.30–6.04 U/mL).

## 4. Discussion

This study is the first to assess the effect of HBOT in conjunction with daptomycin and rifampicin therapy for implant-associated OM caused by *S. aureus* biofilm infection. While HBOT appeared to benefit animals by reducing abscess signs, the primary outcomes (cure rate and bacterial load in bone and on implants) were unaffected by HBOT, both in animals receiving and not receiving antibiotics. Hence, our data revealed no significant benefit of adding HBOT to daptomycin and rifampicin compared to treatment based only on the antibiotics (Figure 2).

Our data contradicts several previous studies. For example, Shandley et al. used an experimental model similar to ours to test the effect of HBOT (80 min of 100% O_2_ at 240 kPa) on bacterial growth in implant-associated OM. The bacterial load (CFU/tibia) of MRSA in infected tibias was significantly higher in HBOT-treated animals, and the authors concluded that HBOT accelerates the growth of *S. aureus* [20]. It could be hypothesized that HBOT could oxygenate hypoxic biofilms and boost bacterial metabolism, which should lead to higher CFU counts in tibias from animals treated with HBOT compared with no HBOT. This hypothesis, however, is not supported by our data since HBOT did not induce statistical difference in the median bacterial load of both implants and bone. This was observed in both the group of animals receiving antibiotics and in the group receiving only NaCl (Figure 2).

It has been found that the effect of two hours of HBOT at 200 kPa was comparable to cephalotin for the treatment of OM [16], and others found that while HBOT alone was inferior to e.g. cefazolin treatment, the combination of cefazolin with HBOT (1 h at 300 kPa) was superior to cefazolin monotherapy [19]. In our study, daptomycin and rifampicin treatment was clearly superior to NaCl + HBOT, while the combination of antibiotics with HBOT did not increase their efficacy. The differences in outcome from these studies may be explained by the choice of antibiotics because some types of antibiotics are more effective against metabolically active cells, while other types are not. For example, β-lactams like ampicillin exert their bactericidal effect by the generation of free hydroxyl radicals via the Fenton reaction, a process requiring molecular oxygen [38]. The bactericidal effect of many antibiotics can only be achieved for metabolically active cells, and this was recently shown by demonstrating that pre-treatment of bacteria with a bacteriostatic antibiotic protects them against the antibacterial effect of several β-lactam, aminoglycoside, and fluoroquinolone antibiotics [39]. Cephalosporins (also β-lactams) are the drugs most commonly used in combination with HBOT [16,19] in previous studies, and the apparent synergy between HBOT and antibiotic therapy could be related to these Fenton reaction-derived free hydroxyl radicals. Daptomycin, however, kills bacteria by depolarizing the cell membrane [40] and its efficacy is unaffected by the metabolic activity of the cell [39]. Hence, an HBOT-mediated boost in metabolic activity is less likely to affect the efficiency of this drug. Retrospectively, it would have been interesting to include additional antibiotic combinations, allowing for a side-by-side comparison of the effect of HBOT on the efficacy of antibiotics that targets the membrane versus biosynthesis processes. We did, however design the study to specifically test daptomycin, as this novel anti-staphylococcal is relatively untested in both clinical and preclinical studies.

HBOT has been proposed to have several beneficial effects upon bone tissue, namely an mobilization of osteoclast to increase resorption of necrotic bone [21]. However, a recent in vivo study on the effect of HBOT on human osteoclast recruitment found that 100% O_2_ at 240 kPa for 90 min daily for either 10 or 25 days reduced osteoclast recruitment and decreased osteoclast-mediated resorption of bone [41]. These findings and those reported in our study contradict previous sentiments on HBOT’s effect on these cell lines [21]. Osteoclast activity was slightly elevated in animals treated with HBOT and antibiotics compared to animals treated solely with antibiotics, yet this did not have any effect on bacterial burden. Whether this has any long-term benefit for bone healing is uncertain, as our animals were euthanized before any such assessment could be made. It is of note that the levels of TRAP 5b in animals treated with NaCl was not different to the animals treated with HBOT and antibiotics, further demonstrating that the changes in TRAP 5b levels are likely of little importance (Figure 4). A similar pattern was observed for P1NP (Figure 3).

Another limitation of our study was the fact that our animal model was not based on a treatment-refractory infection. Future studies on HBOT for implant-associated OM should be conducted on a model for treatment of a refractory biofilm infection, which currently does not exist. Presently, all models of implant-associated OM demonstrate high cure rates when antibiotic combinations include rifampicin. A model of implant-associated OM with a 4-week-old infection was still highly susceptible to both vancomycin + rifampicin and tigecycline + rifampicin combinations, as the infection was eradicated from implants in more than 70% of animals [42]. Commencing treatment in animals with 4 weeks of infection is interesting, as clinical guidelines only recommend antibiotic therapy and debridement for infections arising within the first 3 weeks following implantation [12]. An alternative to increasing the infection duration prior to treatment start is to decrease the efficacy of the antibiotic therapy. This can be done be either shortening the treatment duration or abstaining from rifampicin based treatment. While both possibilities are not without merit, the experimental setup will poorly reflect the clinical setting, and as such interpretation of HBOTs effectiveness against implant-associated OM is difficult. Finally, the option of removing rifampicin could be considered. This will most certainly decrease the overall effectiveness of antibiotic treatment. This, however, would be of less interest to a clinician, as one would always use an antibiotic combination containing rifampicin as is recommended by current guidelines [12,15]. Finally, increasing the duration of HBOT might be required to observe an effect. This has been demonstrated in vitro, where 4 h of HBOT (100% O_2_ at 280 kPa) increased the efficacy of ciprofloxacin treatment of a mature agarose-embedded *Pseudomonas aeruginosa* biofilm compared to ciprofloxacin treatment at ambient pressure [26]. It is notable this study found that 2-h HBOT in combination with ciprofloxacin was comparable to ciprofloxacin at ambient pressure. Whether a similar pattern exists for *S. aureus* and whether animals can tolerate repeated sessions of HBOT with duration of 4 h is unknown.

To our knowledge, this is the first study assessing the effect of HBOT on the efficacy of daptomycin + rifampicin treatment of implant-associated OM. Although HBOT did not increase the cure rate, we found that the daptomycin + rifampicin treatment in itself was very effective, resulting in eradication of implant biofilms in 75% of animals and eradication of adjacent OM in 40% of animals. This is comparable to the effect of vancomycin + rifampicin tested in similar models [27,42]. Daptomycin + rifampicin has only been tested once in an experimental model of of implant-associated OM, and the only outcome was reduction of bacterial load in infected bone, where the cure rate was 100% [32]. As such, further animal studies are required, preferably on clinical isolates. Should daptomycin, in combination with rifampicin, continue to exhibit higher cure rates against different strains of *S. aureus* in direct comparison with vancomycin + rifampicin, the combination would be candidate for a randomized clinical trial. Currently, daptomycin is a second-line drug against acute biofilm infection with both Methicillin-sensitive *Staphylococcus aureus* and MRSA in the orthopedic setting [43]. Based on these results, daptomycin + rifampicin therapy is a promising combination, which should be tested in a clinical setting of *S. aureus* implant-associated OM.

## 5. Conclusions

We found that HBOT did not improve the efficacy of daptomycin + rifampicin treatment of implant-associated OM, and in contrast to previous studies, we found that HBOT did not increase the amount of bacteria in infected tibias, nor did HBOT induce marked changes in bone turnover. However, we found that the combination of daptomycin and rifampicin was very effective against implant-associated osteomyelitis.

## Figures and Tables

**Table 1:** Abscess signs.

	−	+	++
NaCl + HBOT	5	9	6
NaCl	0	0	20
Daptomyicin + rifampicin + HBOT	7	11	2
Daptomyicin + rifampicin	4	12	4
